# Exploring the Antimicrobial Potential of a Novel Phage-Derived Lytic Protein Against *Pseudomonas aeruginosa*

**DOI:** 10.3390/cimb48030318

**Published:** 2026-03-17

**Authors:** Sibongile Mtimka, Kanyane Bridgett Malatji, Patrick Opare Sakyi, Noel David Nogbou, Andrew Munyalo Musyoki, Sipho Mamputha, Lusisizwe Kwezi, Samuel Kojo Kwofie, Ofentse Jacob Pooe, Tsepo Lebiletsa Tsekoa

**Affiliations:** 1 Chemicals Cluster, Council for Scientific and Industrial Research (CSIR), Pretoria 0001, South Africa; 2 Discipline of Biochemistry, School of Life Sciences, University of KwaZulu-Natal, Durban 4001, South Africa; 3 Department of Chemical Sciences, University of Energy and Natural Resources, Sunyani P.O. Box 214, Ghana; 4 Department of Microbiology, School of Medicine, Sefako Makgatho Health Sciences University, Pretoria 0204, South Africa; 5 Biomedical Engineering Department, School of Engineering Sciences, University of Ghana, Legon, Accra Ga488-6615, Ghana; 6 Institute of Applied Science and Technology, University of Ghana, Legon, Accra Ga488-6615, Ghana

**Keywords:** endolysin, antimicrobial, *Pseudomonas aeruginosa*, phage-derived protein, catalytic domain, bacteriophage therapy

## Abstract

The escalation of bacterial resistance to existing antibiotics represents a growing global health challenge, exacerbated by the widespread misuse of antimicrobial agents. As a result, alternative antibacterial strategies are increasingly being explored, including phage-derived lytic proteins. In this study, we report a preliminary characterisation of a novel phage-derived lytic protein identified through computational screening of bacteriophage genome sequences. A putative open reading frame, designated SM07 (1383 bp), was selected from bacteriophage sequences contributed by the University of KwaZulu-Natal to a global phage repository. The gene was synthesised, sub-cloned into the pET-30b(+) vector with an N-terminal histidine tag, and recombinantly expressed in *Escherichia coli* BL-21(AI) cells. The protein was purified using affinity and ion-exchange chromatography. Purified SM07 exhibited in vitro antimicrobial activity against *Pseudomonas aeruginosa*, with a minimum inhibitory concentration of 4 µg/mL, while no significant cytotoxic effects were observed in Vero kidney cells at concentrations substantially above the effective dose. Together, these findings provide initial evidence supporting the antimicrobial potential of SM07 and highlight phage-derived lytic proteins as candidates for further investigation as alternative agents against *P. aeruginosa*-associated infections.

## 1. Introduction

The global rise in antimicrobial resistance (AMR) demands innovative therapeutic approaches. The World Health Organization (WHO) projects that deaths associated with multidrug-resistant infections may reach ten million annually by 2050 and will also have severe consequences for global health and related economic activities [1,2,3,4]. Although this is a global health burden, it is more severe in developing countries [5,6]. The WHO has identified a range of disease-causing bacteria that require immediate attention; this list includes ESKAPE pathogens (*Enterococcus faecium*, *Staphylococcus aureus*, *Klebsiella pneumoniae*, *Acinetobacter baumannii*, *Pseudomonas aeruginosa*, and *Enterobacter* spp.) [7,8]. These pathogens account for various nosocomial infections, especially in infants, the elderly, and immunocompromised individuals [7]. The rate of nosocomial infection in developing countries is almost double that in developed countries [9,10]. Antibiotics have become ineffective due to bacteria gaining resistance via horizontal gene transfer, pathways that allow them to bypass the effects of the antibiotics, or by spontaneous mutations that change the antibiotic target or upregulate efflux pumps [11]. Identifying alternative antimicrobial agents with distinct mechanisms of action is therefore increasingly important.

Among the emerging alternatives, phage-derived lytic proteins (endolysins) have attracted increasing attention as targeted antibacterial agents [12,13]. The targeted action of phage-derived lytic enzymes, which specifically attack components of bacterial cell walls, has positioned them as promising antimicrobial candidates [14,15,16]. Holger et al. (2021) highlighted the clinical pharmacology and potential of phage therapy against multidrug-resistant superbugs [17]. Phage-derived lytic enzymes can be used alone or in combination with existing antibiotics, thereby increasing treatment efficacy. Ongoing clinical pharmacology studies aim to optimise dosing, delivery methods, and safety profiles to translate these enzymes from experimental agents to mainstream therapies in combating resistant bacterial infections [18].

Phage-derived lytic proteins typically comprise one or more catalytic/enzymatic active domains (EADs) and, in many cases, a cell wall-binding domain (CBD) [19,20]. These domains are found on the protein’s N- or C-terminal based on the type of bacteria they target. With computational structural modelling, valuable information can be obtained on the function and behaviour of proteins [21]. Because many endolysins exhibit limited stability under conditions required for crystallographic analysis, computational structural modelling has become an important complementary approach for predicting their structure and function [19,20].

This study aimed to apply sequence-based screening approaches to identify, recombinantly produce, and preliminarily characterise a novel phage-derived lytic protein from a bacteriophage sequence repository, and to evaluate its in vitro antimicrobial activity against selected bacterial pathogens. Endolysins have considerable potential as biotherapeutic agents due to their ability to target and degrade bacterial cell walls. The specificity of endolysins and low likelihood of resistance make them a promising alternative for combating antibiotic-resistant bacteria, offering hope for future medical treatment of antimicrobial resistance.

## 2. Materials and Methods

### 2.1. Phage Database Screening and Sequence Analysis

Phage-derived lytic proteins were screened from the PhagesDB repository (https://phagesdb.org/institutions/KRIT/ accessed on 3 March 2020), focusing on bacteriophage sequences originating from the University of KwaZulu-Natal [22]. Nucleotide sequences were translated using BioEdit 7.2.5 (six-frame translation, minimum ORF length of 100 amino acids, start codon ATG) [23], and additional sequence analyses were performed using CLC Bio Workbench 6 [24]. Homology searches were conducted using National Center for Biotechnology Information basic local alignment search tool (NCBI BLAST) (http://www.ncbi.nlm.nih.gov/BLAST/ accessed on 3 March 2020) [25]. Conserved domains were identified using the NCBI Conserved Domain Database (CDD) (https://www.ncbi.nlm.nih.gov/Structure/cdd/wrpsb.cgi?SEQUENCE accessed on 3 March 2020, updated 23 October 2025).

Signal peptides were predicted using the SignalP 4.1 server, while theoretical molecular weight, isoelectric point (pI), and hydropathy profiles were calculated using the ExPASy Compute pI/Mw (https://web.expasy.org/compute_pi/ accessed on 18 June 2020) and ProScale tools [26]. Structural similarity analysis was performed by aligning the predicted protein sequence with homologous crystallised proteins available in the Protein Data Bank (PDB).

### 2.2. Development of Expression Plasmids by PCR 

The DNA fragment encoding the lysin gene was amplified using the Q5 PCR kit (New England Biolabs, Ipswich, MA, USA) from a synthesised pUC57 construct (GenScript, Piscataway, NJ, USA). Primers were designed to introduce an N-terminal His_6_ tag, an NdeI restriction site at the 5′ end (5′ catatgCACCACCACCACCACCACaccgcgatcattaccc), and a stop codon upstream of the XhoI site at the 3′ end (3′ CTCGAGAATGTTGCCTTTCAG) to prevent incorporation of the vector-encoded C-terminal His_6_ tag. PCR amplification was performed using the following cycling conditions: initial denaturation at 98 °C for 1 min, followed by 24 cycles of denaturation at 98 °C for 30 s, gradient annealing from 55 °C to 69 °C (0.6 °C increments) for 30 s, and extension at 72 °C for 30 s, with a final extension at 72 °C for 5 min. The amplicons were prepared for ligation by subjecting them to a restriction reaction with NdeI and XhoI and ligated into the pET-30b(+) vector to generate the expression construct pET30_Lysin.

### 2.3. Recombinant Protein Production and Purification of Recombinant Proteins

Recombinant SM07 was expressed in Escherichia coli BL21(AI) cells (Thermo Fisher Scientific, Waltham, MA, USA) transformed with the pET30_Lysin construct. A single colony was used to inoculate LB medium supplemented with kanamycin (50 µg/mL; Sigma-Aldrich, St. Louis, MO, USA) and grown overnight at 37 °C with shaking. The culture was diluted 1:100 into fresh LB medium and incubated until an OD_600_ of 0.4–0.6 was reached. Protein expression was induced with 0.1 mM IPTG (Sigma-Aldrich, St. Louis, MO, USA) and 0.2% (*w*/*v*) L-arabinose (Sigma-Aldrich, St. Louis, MO, USA), followed by incubation for 4 h at 37 °C.

Cells were harvested by centrifugation at 10,000 rpm for 10 min at 4 °C and resuspended in bacterial protein extraction reagent (B-PER; Thermo Fisher Scientific, Waltham, MA, USA) supplemented with protease inhibitor cocktail (Sigma-Aldrich, St. Louis, MO, USA). Lysates were clarified by centrifugation, and the soluble fraction was subjected to immobilised metal affinity chromatography (IMAC) using Protino^®^ Ni-TED resin (Macherey-Nagel, Düren, Germany) under native conditions. Bound His_6_-tagged SM07 was eluted with buffer containing 250 mM imidazole (Sigma-Aldrich, St. Louis, MO, USA). Purity and expression were assessed by SDS-PAGE and confirmed by Western blot analysis using an anti-His antibody (Thermo Fisher Scientific, Waltham, MA, USA).

### 2.4. Second Purification Step of the Purified Protein Using Ion Exchange

Following IMAC purification, SM07-containing fractions were dialysed against 20 mM Tris buffer (Sigma-Aldrich, St. Louis, MO, USA), pH 6.5, to remove imidazole and ensure compatibility with ion exchange chromatography. Further purification was performed using an ÄKTA Avanta 150 system (Cytiva, Uppsala, Sweden). Anion exchange chromatography was conducted using CaptoQ resin (Cytiva, Uppsala, Sweden). The column was equilibrated with 5 column volumes (CV) of start buffer (20 mM Tris, pH 6.5), and after sample loading, washed with 5 CV of start buffer. Bound proteins were eluted using a 0–100% linear gradient of elution buffer (20 mM Tris, 1 M NaCl; Sigma-Aldrich, St. Louis, MO, USA, pH 6.5) over 20 CV. Eluted fractions were analysed by SDS-PAGE and confirmed by Western blotting prior to downstream characterisation.

### 2.5. Mass Spectrometry Identification of Purified Lysin via In-Gel Trypsin Digest

The protein band corresponding to purified SM07 was excised from a 12% SDS-PAGE gel and subjected to in-gel digestion. Gel pieces were destained using ammonium bicarbonate (Sigma-Aldrich, St. Louis, MO, USA) and methanol (Sigma-Aldrich, St. Louis, MO, USA), followed by reduction and alkylation. Proteins were digested overnight at 37 °C using sequencing-grade trypsin (Promega, Madison, WI, USA). Peptides were extracted, dried under vacuum, and resuspended prior to analysis by liquid chromatography–electrospray ionisation tandem mass spectrometry (LC–ESI–MS/MS). Data were analysed using ProteinPilot™ software 5.0 (SCIEX, Framingham, MA, USA) against relevant protein databases. Peptide matches were accepted based on confidence scores ≥ 95% and mapped to the predicted SM07 sequence.

### 2.6. Antimicrobial Susceptibility Test

The antibacterial activity of SM07 was initially evaluated using an agar well diffusion assay adapted from the method of Reeves et al. [27]. Mueller–Hinton agar (MHA) plates (100 mm) were prepared according to manufacturer instructions. Overnight bacterial cultures were adjusted to a turbidity equivalent to a 0.5 McFarland standard [28]. in sterile saline, in alignment with Clinical and Laboratory Standards Institute (CLSI) recommendations for antimicrobial susceptibility testing. Standardised bacterial suspensions were evenly spread onto MHA plates using sterile swabs. Wells were created using a sterile borer, and purified SM07 was added to each well. Plates were incubated at 37 °C for 18–24 h, and zones of inhibition were measured. All experiments were conducted in triplicate. Positive control antibiotics were selected based on strain-specific CLSI guidance: Cefixime (30 µg) for *Acinetobacter baumannii* BAA747; Augmentin (30 µg) for *Enterococcus faecalis* ATCC51299 and Escherichia coli ATCC35218; and Cefotaxime (30 µg) for *Staphylococcus aureus* ATCC977BA and *Pseudomonas aeruginosa* ATCC27853. Negative controls included phosphate-buffered saline (PBS), empty pET-30b(+) vector lysate, and BL21(AI) expression host lysate.

Minimum inhibitory concentration (MIC) values were determined for susceptible strains using the broth microdilution method described by Eloff [29]. MIC was defined as the lowest concentration at which no visible bacterial growth was observed following incubation.

### 2.7. Lysin (SM07) Cytotoxicity Studies in Normal Vero Kidney Cells

Cytotoxicity of purified SM07 was evaluated using Vero kidney epithelial cells. Cells were seeded at a density of 1 × 10^4^ cells per well in 96-well plates and incubated for 24 h at 37 °C under 5% CO_2_ and 95% humidity to allow adherence. Two-fold serial dilutions of SM07 were prepared in culture medium, starting at 62.5 µg/mL. Following medium removal, 100 µL of each SM07 dilution was added to the corresponding wells. Phosphate-buffered saline (PBS)-treated cells served as negative controls. All treatments were performed in triplicate and incubated for 48 h.

Cell viability was assessed using the MTT assay. Briefly, 25 µL of MTT solution (5 mg/mL; Sigma-Aldrich, St. Louis, MO, USA) was added to each well and incubated for 3 h at 37 °C to allow formation of formazan crystals. The MTT solution was removed, and 100 µL dimethyl sulfoxide (DMSO; Sigma-Aldrich, St. Louis, MO, USA) was added to dissolve the formazan product. Absorbance was measured at 620 nm using a Tecan Infinite F500 microplate reader (Tecan, Männedorf, Switzerland). Cell viability was expressed relative to untreated control cells. Statistical analysis was performed to assess differences between treated and control groups.

## 3. Results

### 3.1. Sequence Analysis and Cloning Lysin (SM07)

**Figure 1:**
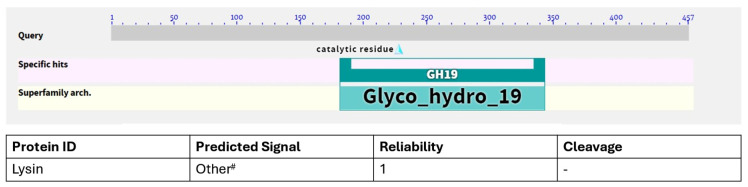
Graphic summary of putative conserved domains detected in lysin (endolysins). The domain within lysin shows a relationship to the lysozyme-like superfamily, which plays a role in cleaving PG. The following is the Signal Peptide Prediction Report for lysin. # Neither a signal sequence nor a membrane-spanning part is expected at the N-terminus of the protein.

**Figure 2:**
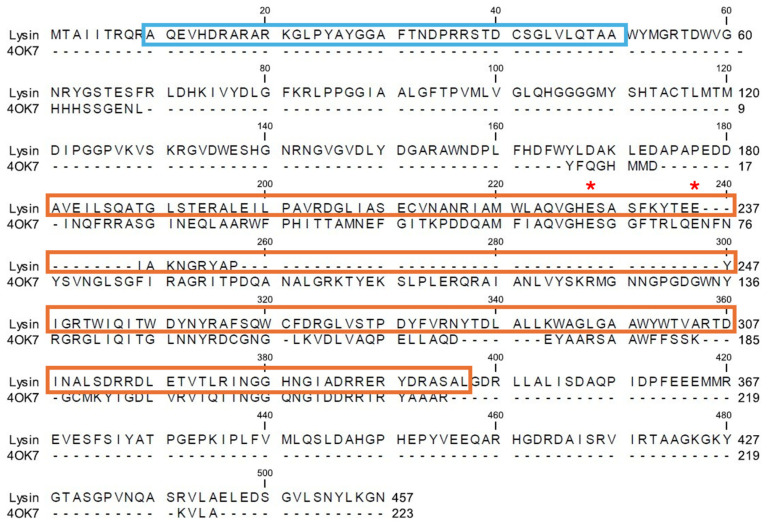
Modular composition of the full-length sequence of the phage-derived protein (SM07) aligned with the amino acid sequence of the SPN1S endolysins (4OK7) with catalytic residues highlighted with red asterisks and putative N-terminal CBD (Blue) and C-terminal catalytic domain (Orange).

A candidate lysin, designated SM07, was identified through sequence-based screening of a bacteriophage database, leveraging the established antimicrobial potential of phage-derived lytic proteins. From the translated open reading frames (ORFs), SM07 (1383 bp) was selected for further analysis based on the presence of conserved domains consistent with phage-derived lytic enzymes (Figure 1). Other candidate ORFs were reserved for future investigation. Comparative structural modelling against proteins deposited in the Protein Data Bank (PDB) revealed structural similarity to the SPN1S endolysin of *Salmonella typhimurium* (PDB: 4OK7), with 25.24% sequence identity across 34% query coverage. Additional similarity was observed with GH19 chitinases (PDB entries 2CJL, 5H7T, and 4MCK). Sequence alignment suggested the presence of a putative catalytic domain and a potential cell wall–binding domain, as illustrated in Figure 2. Based on these analyses, the SM07 coding sequence was subcloned into the pET-30b(+) expression vector under the T7 promoter, incorporating an N-terminal His_6_ tag to facilitate purification.

### 3.2. Recombinant Expression of the Phage-Derived Lytic Protein (SM07)

**Figure 3:**
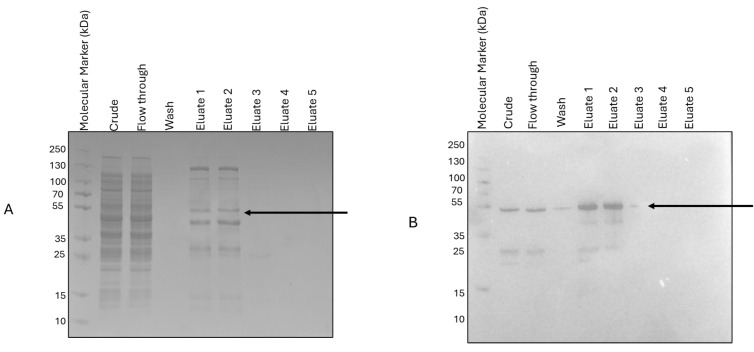
First-step purification was performed using IMAC-NiTED resin to be used in the second-step purification using ion exchange. (**A**) SDS-PAGE and (**B**) Western Blot analysis of purification.

**Figure 4:**
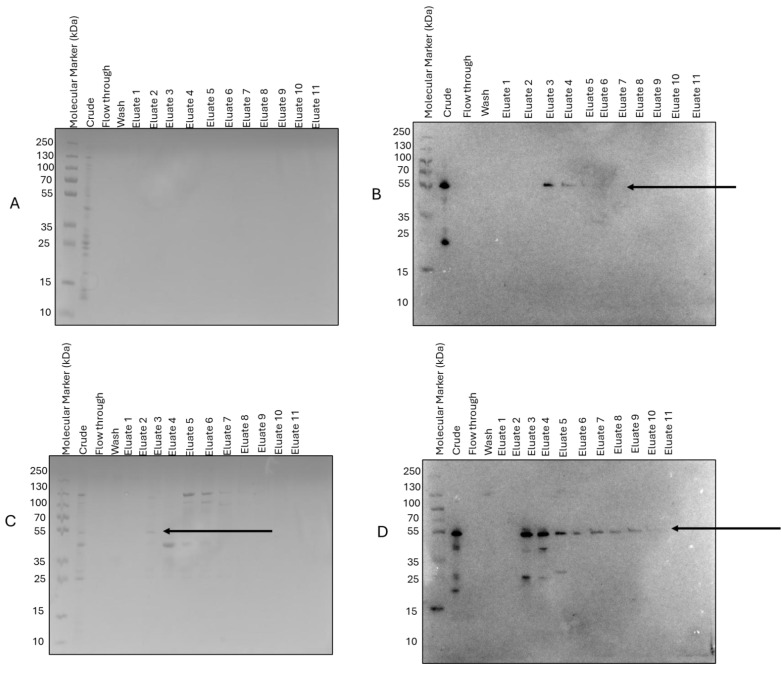
Second step purification using ion exchange. (**A**) SDS-PAGE and (**B**) Western Blot analysis of CaptoDEAE purification after immobilised metal ion affinity chromatography (IMAC) purification, and (**C**,**D**) SDS-PAGE and Western Blot analyses of CaptoQ purification.

SM07 expression was successfully achieved using BL-21(AI) cells in LB medium at 37 °C with agitation at 200 rpm, with a final induction concentration of 0.1 mM IPTG and 0.2% arabinose; purification was achieved using Ni-TED immobilized metal affinity chromatography resin (Figure 3). This purity was subsequently refined by ion-exchange chromatography to eliminate co-purifying proteins. Our findings notably favoured anion exchange chromatography, specifically CaptoQ resin, over CaptoDEAE (Figure 4). The corresponding band on Figure 4C was cut for MS analysis.

### 3.3. Identity Confirmation of the SM07 Protein Using Mass Peptide Fingerprinting

**Figure 5:**
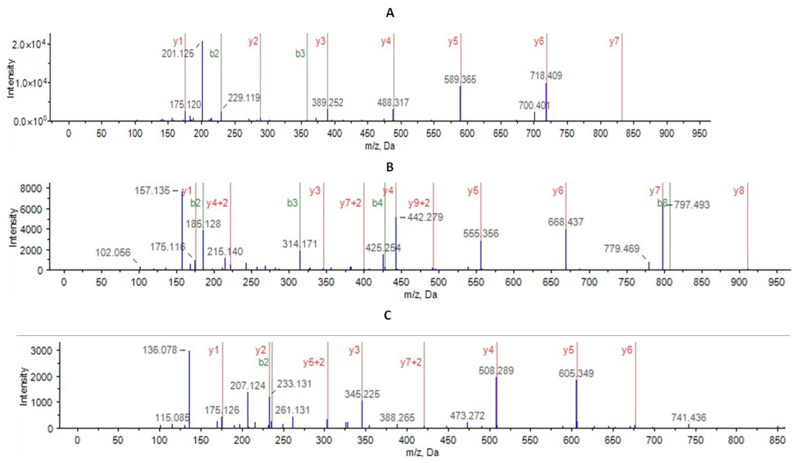
Peptide Mass fingerprinting of phage-derived lytic protein using Protein Pilot 5.0 revealed endolysin with the three peptides (**A**) (DLETVTLR), (**B**) (ALEILPAVR) and (**C**) (YAPYIGR). The schematic is from the ionization spectra of the identified endolysin.

The protein band corresponding to purified SM07 (Figure 4C) was excised from SDS-PAGE and subjected to in-gel tryptic digestion followed by LC–ESI–MS/MS analysis. Peptide mass fingerprinting identified six peptide fragments, three of which matched the predicted SM07 sequence with confidence scores ≥ 95%. The identified peptide sequences (DLETVTLR, ALEILPAVR, and YAPYIGR) are shown in Figure 5. The detected peptides mapped to regions within the predicted lysin sequence, thereby confirming the identity of the expressed protein. While sequence coverage was limited, the high-confidence peptide matches, together with the expected molecular weight and Western blot detection of the His_6_ tag, support successful recombinant expression and purification of SM07.

### 3.4. Anti-Microbial Screening of SM07 Produced Recombinantly

The antimicrobial activity of purified SM07 was evaluated against representative ESKAPE pathogens (Table 1). Among the strains tested, detectable antibacterial activity was observed only against *Pseudomonas aeruginosa* ATCC27853. No measurable zones of inhibition were observed for *Acinetobacter baumannii*, *Escherichia coli*, *Staphylococcus aureus*, or *Enterococcus faecalis* under the conditions tested. For *P. aeruginosa*, purified SM07 produced a clear zone of inhibition in the agar well diffusion assay. The minimum MIC, determined using the broth microdilution method, was 4 µg/mL. Negative controls, including PBS, empty pET-30b(+) vector lysate, and BL21(AI) expression host lysate, showed no inhibitory activity against any strain tested.

Crude lysate preparations of SM07 did not demonstrate detectable antibacterial activity. This may reflect lower effective concentrations of active protein in the crude extract or interference from host-derived proteins. Activity was observed without the use of an outer membrane-permeabilising agent. While many reported endolysins targeting Gram-negative bacteria require membrane destabilisation for activity, SM07 exhibited measurable inhibition of *P. aeruginosa* under the assay conditions employed. Further studies will be required to clarify the mechanism underlying this activity.

### 3.5. Cytotoxicity Studies

**Figure 6:**
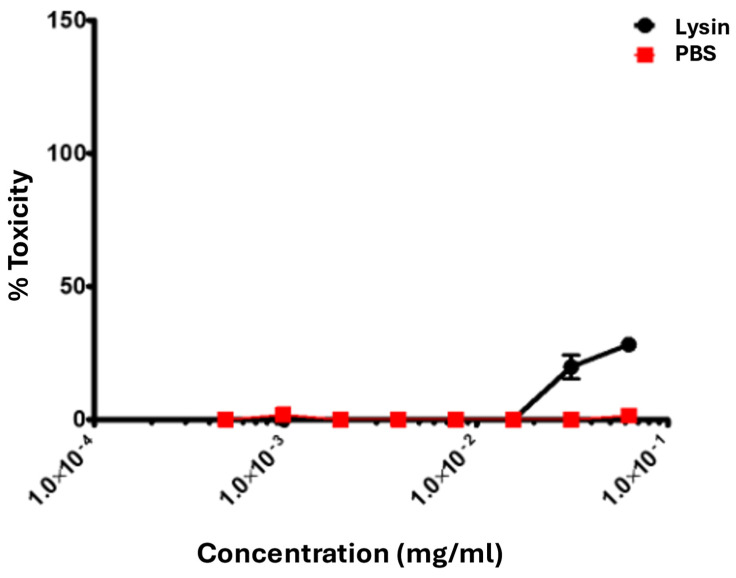
Lysin toxicity evaluation. The data displayed are the mean and standard deviations of three experiments. The y-axis indicates the percent toxicity level, while the x-axis indicates the concentration levels of the lysin treatment (black) and the PBS control (red) in ascending order from left to right.

Our data demonstrated that SM07 exhibited no significant toxicity against Vero cells. Even at the highest concentration tested (62.5 µg/mL, ~15× the MIC of *P. aeruginosa*), SM07 did not cause significant toxicity in Vero cells (Figure 6), suggesting that the protein is relatively safe at concentrations well above the effective dose.

## 4. Discussion

Phage-derived lytic proteins are increasingly investigated as alternatives or complements to conventional antibiotics in response to escalating antimicrobial resistance [4,11,30]. Using sequence-based screening of publicly available bacteriophage datasets, we identified several candidate open reading frames encoding putative lytic proteins. Among these, SM07 was selected for recombinant production and preliminary functional evaluation.

Bioinformatic analysis indicated that SM07 contains conserved motifs associated with the lysozyme-like superfamily and lacks a predicted signal peptide. Structural comparison with deposited PDB entries suggested similarity to the SPN1S endolysin and related GH19 chitinases. While sequence identity was moderate, these findings supported further experimental characterisation.

Alignment with the SPN1S endolysin structure (PDB: 4OK7) [31] suggested the presence of a putative catalytic domain and a region consistent with a potential cell wall-binding domain. Conserved glutamate residues (Glu228 and Glu237) were identified within the predicted catalytic region. These structural and catalytic interpretations are based on in silico modelling and sequence homology and therefore should be considered hypothesis-generating rather than experimentally validated. These residues are consistent with catalytic motifs reported in lysozyme-like endolysins that cleave β-(1-4)-glycosidic bonds in peptidoglycan [32]. Endolysins with similar modular architectures have been widely reported to exhibit antibacterial activity through targeted peptidoglycan degradation [33]. While sequence similarity was moderate, these observations support the hypothesis that SM07 may possess muralytic activity; however, direct biochemical validation of catalytic function will be required.

Recombinant expression of SM07 in BL21(AI) cells yielded soluble protein, although overall expression levels were modest. Following IMAC, further purification was performed using anion exchange chromatography to improve protein purity. Ion exchange chromatography is widely used for the separation of recombinant proteins based on charge properties and is well established in biopharmaceutical purification workflows [34,35]. In the present study, strong anion exchange chromatography (CaptoQ) provided improved separation compared to weaker anion exchange conditions (CaptoDEAE), consistent with differences in resin functional groups and binding strength [36,37]. The choice of purification strategy was guided by the predicted isoelectric point of SM07 and aimed at obtaining material suitable for downstream characterisation. Peptide mass fingerprint analysis confirmed the identity of purified SM07. This approach is widely used for protein identification and validation following electrophoretic separation [38]. High-confidence peptide matches (≥95%) mapped to the predicted SM07 sequence and were consistent with the expected molecular weight and Western blot detection of the His_6_ tag. While overall sequence coverage was limited, the combined analytical evidence supports successful recombinant expression and purification of SM07. The biological activity assessment of the SM07 protein was tested against ESKAPE pathogens for antimicrobial potential. Purified SM07 exhibited measurable in vitro activity against Pseudomonas aeruginosa ATCC27853, while no detectable inhibition was observed for the other tested strains under the assay conditions employed. This apparent selectivity may reflect species-specific differences in cell wall architecture, outer membrane permeability, or assay sensitivity.

SM07 is predicted to possess a modular architecture comprising a putative catalytic domain and a region consistent with a potential cell wall–binding domain (CBD). CBDs are typically associated with enhanced specificity through targeted recognition of peptidoglycan motifs and have been extensively characterised in modular endolysins [39,40]. Activity was observed in the absence of an outer membrane-permeabilising agent.

Activity was observed in the absence of an outer membrane-permeabilising agent. Although many reported endolysins targeting Gram-negative bacteria require membrane destabilisation to access peptidoglycan, SM07 demonstrated inhibition under the conditions tested. This contrasts with engineered lysin approaches, including Artilysins and peptide-fusion constructs designed to enhance outer membrane penetration, and therefore positions SM07 as a naturally occurring candidate exhibiting measurable activity without synthetic modification. The mechanism underlying this activity remains to be elucidated and warrants further structural and functional investigation.

Inhibition of *P. aeruginosa*, an organism known to cause infections in the blood, lungs, urinary tract, and gastrointestinal tract [41], supports further investigation of SM07 as a potential candidate for targeted antimicrobial development. A study by Fischetti [12] demonstrated that endolysins are less active against Gram-negative bacteria, but SM07 showed good inhibition, even showing moderate but statistically significant inhibition against another Gram-negative bacterium. Crude lysate preparations did not demonstrate detectable antibacterial activity, likely due to lower effective concentrations of active protein or interference from host-derived components.

These observations highlight their potential; phage-derived lysins face several challenges for therapeutic application. As protein biologics, they can exhibit limited stability, susceptibility to proteolytic degradation, and formulation constraints. In Gram-negative bacteria, delivery to the peptidoglycan layer remains a recognised barrier due to the outer membrane. In addition, expression yield, scalability, and purification efficiency may impact large-scale production. These limitations can be addressed through protein engineering to improve stability and activity, formulation optimisation, and combination approaches with membrane-permeabilising agents or conventional antibiotics, which have been shown to enhance lysin efficacy.

Assessing preliminary cytotoxicity is vital for evaluating safety, biocompatibility, and therapeutic benefits of substances, especially in biomedical and pharmaceutical applications. Studies on the cytotoxicity of phage-derived proteins show that some of these proteins can be toxic [42]. Preliminary cytotoxicity assessment in Vero cells indicated no significant reduction in cell viability at concentrations substantially exceeding the MIC against *P. aeruginosa*. These findings are consistent with prior reports demonstrating that phage-derived lysins generally exhibit specificity for bacterial peptidoglycan and limited effects on mammalian cells [43]. In contrast, certain conventional antibiotics, including aminoglycosides, are associated with dose-dependent cytotoxicity in mammalian cell systems [44]. Broader cytotoxicity profiling across additional cell types and in vivo models will be required to further define the safety profile of SM07.

Several limitations should be acknowledged. The antimicrobial activity was assessed under controlled laboratory conditions using reference strains, and broader strain panels, clinical isolates, and time–kill kinetics were not evaluated in the present study. In addition, structural modelling was predictive in nature and did not include experimental structural validation. These findings therefore represent a preliminary characterisation of SM07 and provide a foundation for further investigation. Future work will include kinetic lysis assays, broader strain panels including clinical isolates, and biochemical validation of muralytic activity to further define the mechanistic and therapeutic potential of SM07.

In summary, the identification and preliminary characterisation of SM07 demonstrate measurable in vitro antimicrobial activity against *Pseudomonas aeruginosa* under defined laboratory conditions. Bioinformatic analyses suggest the presence of conserved structural features consistent with phage-derived lytic enzymes, and cytotoxicity assessment in Vero cells indicated no significant reduction in viability at concentrations exceeding the observed MIC. While additional structural, mechanistic, and in vivo investigations are required, the present study expands the catalogue of phage-derived lytic proteins and provides a foundation for further exploration of SM07 as a potential antimicrobial scaffold.

## Figures and Tables

**Table 1:** Antimicrobial activity screening against ESKAPE Pathogens.

Strain	Control Antibiotic	Test Samples	Activity	MIC
*Acinetobacter baumannii* BAA747	Cefixime 30 μg	Phosphate Buffer (PBS, negative control 1)	R’	ND
		Empty vector pET-30b (+) vector (negative control 2)	R’	ND
		Expression strain BL-21 (AI) (negative control 3)	R’	ND
		Clarified Crude Lysate of SM07	R	ND
		Purified SM07	R	ND
ESCCO ATCC 25922	Augmentin 30 μg	Phosphate Buffer (PBS, negative control 1)	R’	ND
		Empty pET-30b(+) vector (negative control 2)	R’	ND
		Expression strain BL-21 (AI) (negative control 3)	R’	ND
		Clarified Crude Lysate of SM07	R	ND
		Purified SM07	R	ND
*Staphylococcus aureus* ATCC977BA	Cefotaxime 30 µg	Phosphate Buffer (PBS, negative control 1)	R’	ND
		Empty pET-30b(+) vector (negative control 2)	R’	ND
		Expression strain BL-21 (AI) (negative control 3)	R’	ND
		Clarified Crude Lysate of SM07	R	ND
		Purified SM07	R	ND
*Enterococcus faecalis* ATCC51299	Augmentin 30 μg	Phosphate Buffer (PBS, negative control 1)	R’	ND
		Empty pET-30b(+) vector (negative control 2)	R’	ND
		Expression strain BL-21 (AI) (negative control 3)	R’	ND
		Clarified Crude Lysate of SM07	R	ND
		Purified SM07	R	ND
*Pseudomonas aeruginosa* ATCC27853	Cefotaxime 30 µg	Phosphate Buffer (PBS, negative control 1)	R’	ND
		Empty pET-30b(+) vector (negative control 2)	R’	ND
		Expression strain BL-21 (AI), (negative control 3)	R’	ND
		Clarified Crude Lysate of SM07	R	ND
		Purified SM07	Y	4 µg/mL
*Escherichia coli* ATCC35218	Augmentin 30 μg	Phosphate Buffer (PBS, negative control 1)	R’	ND
		Empty pET-30b(+) vector (negative control 2)	R’	ND
		Expression strain BL-21 (AI), (negative control 3)	R’	ND
		Clarified Crude Lysate of SM07	R	ND
		Purified SM07	R	ND

R—Resistant, R’—Negative Control. ND—Not Determined. Y—Yes.

## Data Availability

The raw data supporting the conclusions of this article will be made available by the authors on request.

## References

[1] Criscuolo E., Spadini S., Lamanna J., Ferro M., Burioni R. (2017). Bacteriophages and Their Immunological Applications against Infectious Threats. J. Immunol. Res..

[2] Górski A., Miedzybrodzki R., Borysowski J. (2019). Phage Therapy: A Practical Approach.

[3] World Health Organisation (2019). International Organizations Unite on Critical Recommendations to Combat Drug-Resistant Infections and Prevent a Staggering Number of Deaths Each Year.

[4] Aslam B., Arshad M.I., Aslam M.A., Muzammil S., Siddique A.B., Yasmeen N., Khurshid M., Rasool M., Ahmad M., Rasool M.H. (2021). Bacteriophage Proteome: Insights and Potentials of an Alternate to Antibiotics. Infect. Dis. Ther..

[5] Tadesse B.T., Ashley E.A., Ongarello S., Havumaki J., Wijegoonewardena M., González I.J., Dittrich S. (2017). Antimicrobial resistance in Africa: A systematic review. BMC Infect. Dis..

[6] Ventola C.L. (2015). The Antibiotic Resistance Crisis: Part 1: Causes and threats. Pharm. Ther..

[7] Bhatia P., Sharma A., George A.J., Anvitha D., Kumar P., Dwivedi V.P., Chandra N.S. (2021). Antibacterial activity of medicinal plants against ESKAPE: An update. Heliyon.

[8] Mulani M.S., Kamble E.E., Kumkar S.N., Tawre M.S., Pardesi K.R. (2019). Emerging Strategies to Combat ESKAPE Pathogens in the Era of Antimicrobial Resistance: A Review. Front. Microbiol..

[9] Khan H.A., Baig F.K., Mehboob R. (2017). Nosocomial infections: Epidemiology, prevention, control and surveillance. Asian Pac. J. Trop. Biomed..

[10] Abubakar U., Amir O., Rodríguez-Baño J. (2022). Healthcare-associated infections in Africa: A systematic review and meta-analysis of point prevalence studies. J. Pharm. Policy Pract..

[11] Mtimka S., Pillay P., Kwezi L., Pooe O.J., Tsekoa T.L. (2024). An Exploratory Review of the Potential of Lytic Proteins and Bacteriophages for the Treatment of Tuberculosis. Microorganisms.

[12] Fischetti V.A. (2018). Development of Phage Lysins as Novel Therapeutics: A Historical Perspective. Viruses.

[13] Lin D.M., Koskella B., Lin H.C. (2017). Phage therapy: An alternative to antibiotics in the age of multi-drug resistance. World J. Gastrointest. Pharmacol. Ther..

[14] Fernandes S., São-José C. (2016). More than a hole: The holin lethal function may be required to fully sensitize bacteria to the lytic action of canonical endolysins. Mol. Microbiol..

[15] Gerstmans H., Criel B., Briers Y. (2018). Synthetic biology of modular endolysins. Biotechnol. Adv..

[16] Torres-Barceló C., Hochberg M.E. (2016). Evolutionary Rationale for Phages as Complements of Antibiotics. Trends Microbiol..

[17] Holger D., Kebriaei R., Morrisette T., Lev K., Alexander J., Rybak M. (2021). Clinical Pharmacology of Bacteriophage Therapy: A Focus on Multidrug-Resistant *Pseudomonas aeruginosa* Infections. Antibiotics.

[18] Schmelcher M., Donovan D.M., Loessner M.J. (2012). Bacteriophage endolysins as novel antimicrobials. Future Microbiol..

[19] Cernooka E., Rumnieks J., Zrelovs N., Tars K., Kazaks A. (2022). Diversity of the lysozyme fold: Structure of the catalytic domain from an unusual endolysin encoded by phage Enc34. Sci. Rep..

[20] Low L.Y., Yang C., Perego M., Osterman A., Liddington R.C. (2005). Structure and Lytic Activity of a Bacillus anthracis Prophage Endolysin. J. Biol. Chem..

[21] Haas J., Roth S., Arnold K., Kiefer F., Schmidt T., Bordoli L., Schwede T. (2013). The Protein Model Portal—A comprehensive resource for protein structure and model information. Database.

[22] Russell D.A., Hatfull G.F. (2017). PhagesDB: The actinobacteriophage database. Bioinformatics.

[23] Hall T.A. (1999). BioEdit: A user-friendly biological sequence alignment program for Windows 95/98/NT. Nucleic Acids Symp. Ser..

[24] Scholz M., Lo C.-C., Chain P.S.G. (2014). Improved Assemblies Using a Source-Agnostic Pipeline for MetaGenomic Assembly by Merging (MeGAMerge) of Contigs. Sci. Rep..

[25] Altschul S.F., Gish W., Miller W., Myers E.W., Lipman D.J. (1990). Basic local alignment search tool. J. Mol. Biol..

[26] Gasteiger E., Hoogland C., Gattiker A., Duvaud S., Wilkins M.R., Appel R.D., Bairoch A. (2005). Protein Identification and Analysis Tools on the ExPASy Server. The Proteomics Protocols Handbook; Springer Protocols Handbooks.

[27] Seymour S.L., Hunter C.L. (2015). ProteinPilot^TM^ Software Overview: High Quality. Depth Protein Identification and Protein Expression Analysis.

[28] Mcfarland J. (1907). The Nephelometer: An Instrument for Estimating the Number of Bacteria in Suspensions Used for Calculating the Opsonic Index and for Vaccines. J. Am. Med. Assoc..

[29] Eloff J.N. (1998). A Sensitive and Quick Microplate Method to Determine the Minimal Inhibitory Concentration of Plant Extracts for Bacteria. Planta Medica.

[30] Gondil V.S., Harjai K., Chhibber S. (2020). Endolysins as emerging alternative therapeutic agents to counter drug-resistant infections. Int. J. Antimicrob. Agents.

[31] Park Y., Lim J., Kong M., Ryu S., Rhee S. (2014). Structure of bacteriophage SPN1S endolysin reveals an unusual two-module fold for the peptidoglycan lytic and binding activity. Mol. Microbiol..

[32] Schmitz J.E., Schuch R., Fischetti V.A. (2010). Identifying Active Phage Lysins through Functional Viral Metagenomics. Appl. Environ. Microbiol..

[33] Luong T., Salabarria A.-C., Roach D.R. (2020). Phage Therapy in the Resistance Era: Where Do We Stand and Where Are We Going?. Clin. Ther..

[34] Cummins P., Rochfort K., O’Connor B.F. (2017). Ion-Exchange Chromatography: Basic Principles and Application. Protein Chromatography: Methods and Protocols.

[35] Tripathi N.K. (2016). Production and Purification of Recombinant Proteins from *Escherichia coli*. ChemBioEng Rev..

[36] Fekete S., Beck A., Veuthey J., Guillarme D. (2015). Ion-exchange chromatography for the characterization of biopharmaceuticals. J. Pharm. Biomed. Anal..

[37] Blaschke T., Werner A., Hasse H. (2013). Microcalorimetric study of the adsorption of native and mono-PEGylated bovine serum albumin on anion-exchangers. J. Chromatogr. A.

[38] Hamza M., Ali A., Khan S., Ahmed S., Attique Z., Rehman S.U., Khan A., Ali H., Rizwan M., Munir A. (2020). nCOV-19 peptides mass fingerprinting identification, binding, and blocking of inhibitors flavonoids and anthraquinone of *Moringa oleifera* and hydroxychloroquine. J. Biomol. Struct. Dyn..

[39] Walmagh M., Briers Y., Dos Santos S.B., Azeredo J., Lavigne R. (2012). Characterization of Modular Bacteriophage Endolysins from Myoviridae Phages OBP, 201φ2-1 and PVP-SE1. PLoS ONE.

[40] Schmelcher M., Shabarova T., Eugster M.R., Eichenseher F., Tchang V.S., Banz M., Loessner M.J. (2010). Rapid Multiplex Detection and Differentiation of *Listeria* Cells by Use of Fluorescent Phage Endolysin Cell Wall Binding Domains. Appl. Environ. Microbiol..

[41] Yamada N., Nishida T., Chikama T.I. (2009). *Pseudomonas aeruginosa* Infections. Jpn. J. Clin. Ophthalmol..

[42] Ajuebor J., McAuliffe O., O’Mahony J., Ross R.P., Hill C., Coffey A. (2016). Bacteriophage Endolysins and their Applications. Sci. Prog..

[43] Schuch R., Nelson D., Fischetti V.A. (2002). A bacteriolytic agent that detects and kills Bacillus anthracis. Nature.

[44] Kovacik A., Tvrda E., Jambor T., Fulopova D., Kovacikova E., Hleba L., Kołodziejczyk Ł.M., Hlebova M., Gren A., Massanyi P. (2020). Cytotoxic effect of aminoglycoside antibiotics on the mammalian cell lines. J. Environ. Sci. Health Part A.

